# Serum concentrations of dihydrotestosterone are associated with symptoms of hypogonadism in biochemically eugonadal men

**DOI:** 10.1007/s40618-021-01561-0

**Published:** 2021-04-03

**Authors:** A. Sansone, S. Kliesch, M. Dugas, R. Sandhowe-Klaverkamp, A. M. Isidori, S. Schlatt, M. Zitzmann

**Affiliations:** 1 Center of Reproductive Medicine and Andrology, Institute of Reproductive and Regenerative Biology, Münster, Germany; 2 Chair of Endocrinology and Medical Sexology (ENDOSEX), Department of Systems Medicine, University of Rome Tor Vergata, via Montpellier 1, 00133 Rome, Italy; 3 Institute of Medical Informatics, University of Münster, Münster, Germany; 4 Department of Experimental Medicine, Sapienza University of Rome, Rome, Italy

**Keywords:** Testosterone, Male hypogonadism, Erectile dysfunction, Dihydrotestosterone, International Index of Erectile Function, Aging Males Symptoms scale

## Abstract

**Purpose:**

Symptoms of hypogonadism are often reported by subjects with normal serum testosterone (T) levels. We aimed to assess the association between clinical symptoms in andrological outpatients and sex steroids levels.

**Methods:**

This is a retrospective cross-sectional cohort study in an Academic clinic and research unit. International Index of Erectile Function (IIEF, EF domain) and Aging Males Symptoms scale (AMS) questionnaires were completed by 635 and 574 men, respectively (mean age: 47.3 ± 13.9 and 47.4 ± 13.8 years, *p* = 0.829), free of interfering medications with complaints possibly related to hypogonadism.

**Results:**

Serum total/free T as well as dihydro-T (DHT) was associated with IIEF-EF and AMS scores in the overall population using univariate analyses. Multivariate approaches revealed DHT concentrations in subjects with normal T levels (*n* = 416, Total T > 12 nmol/L) to be significant predictors of AMS scores. A 0.1 nmol/l serum DHT increase within the eugonadal range was associated with a 4.67% decrease in odds of having worse symptoms (*p* = 0.011). In men with biochemical hypogonadism (Total T < 12 nmol/L), total and free T rather than DHT were associated with AMS results. This association was not found for IIEF-EF scores. Indirect effects of age and BMI were seen for relations with hormone concentrations but not questionnaire scores.

**Conclusion:**

DHT can be associated with symptoms of hypogonadism in biochemically eugonadal men. Serum DHT measurement might be helpful once the diagnosis of hypogonadism has been ruled out but should not be routinely included in the primary diagnostic process.

**Supplementary Information:**

The online version contains supplementary material available at 10.1007/s40618-021-01561-0.

## Introduction

Treatment of male hypogonadism has been one of the most controversial topics in endocrinology during the last few years. Several studies have rekindled scientific interests on the side effects and benefits of testosterone (T) administration [1] and have had their coronation in the Testosterone Trials, a series of seven placebo-controlled, double-blind trials in elderly hypogonadal subjects [2]. Most of the reported side effects of T administration had occurred in subjects with high baseline serum T—i.e., subjects who should not have received any kind of androgen replacement therapy—in the hope of improving sexual health and/or physical function. This has led to an FDA warning allowing the use of T only in cases of confirmed low T levels and only in “men with disorders of the testicles, pituitary gland or brain that cause hypogonadism”, as to avoid “attempts to relieve symptoms in men who have low T for no apparent reason other than aging”. Apparently, this addresses men with so-called functional hypogonadism, a condition associated with comorbidities such as obesity/type 2 diabetes mellitus. The T-Trials were performed in elderly men with functional hypogonadism and demonstrated benefits in several dimensions of symptoms [2]. Accordingly, guidelines for the treatment of men with functional hypogonadism have recently been published [3].

Signs and symptoms associated with hypogonadism [4–6] are largely variable: the most common ones, such as fatigue, impaired erectile function and reduced sexual drive, might be the result of several other conditions and are often disregarded as “common features of aging” by patients and clinicians alike [7, 8]. However, signs and symptoms of hypogonadism are mandatory for diagnosis, together with biochemical confirmation of T deficiency [4–6, 9], and should not be overlooked. Testosterone treatment should be considered following diagnosis confirmation, to improve sexual and non-sexual symptoms of hypogonadism [10, 11].

Androgen action is mediated through the androgen receptor (AR); changes in its structure lead to different clinical phenotypes, ranging from complete androgen insensitivity syndrome (CAIS) to apparently normal males [12]. Polymorphisms of the AR gene CAG repeats have also been associated with symptoms of aging in men, namely those of psychological nature [13] but also worse sexual function [14, 15]. While T is undoubtedly the most important androgen, it is worth remembering that the AR also binds its metabolite, 5α-dihydrotestosterone (DHT). T is irreversibly converted to DHT by the microsomal enzyme 5α-reductase, which exists in 3 isoforms present in multiple tissues of the male body (type I: in brain, liver, muscle, skin and prostate; type II: in epididymis, hair follicles, liver, prostate and seminal vesicles; type III: in brain, heart, lung, pancreas, colon, stomach, liver, muscle, prostate and testes).

This leads to measurable serum levels of DHT, which, at least to a certain extent, reflect its biological activity in the overall male organism [16].

Compared to DHT, T has a twofold lower affinity to the AR and a fivefold faster dissociation rate [12], which is largely compensated by significantly higher serum concentrations. While T is secreted under the direct stimulus of pituitary LH, in turn secreted following hypothalamic GnRH stimulus [17, 18], the only regulating factor for DHT conversion is the expression of the 5-alpha-reductase genes and the activity of the isoforms of the transcribed enzyme. In hair follicles, increased expression patterns for both 5αR2 and AR have been associated with androgenetic alopecia [19] and treatment of androgenetic alopecia with 5α-reductase inhibitors (5ARIs) such as finasteride is a definite proof of the involvement of DHT in clinical manifestations of androgen activity as well as side effects of 5ARIs (such as impaired sexual function and more frequent psychological and cognitive complaints), putatively inhibiting especially the cerebral 5αR3 isomer [20–24].

Mutations in the 5αR2-gene lead to the severe phenotype of pseudohermaphoroditism [25–28]. Vice versa, treatment of symptoms of classical hypogonadism might be possible using pure DHT [29], this especially being the case in regard to male sexual function [30].

Nevertheless, it should be debated that a non-aromatizable androgen might have a long-term negative impact on bone integrity.

Based on these assumptions, we hypothesize that in some patients presenting with classical signs and symptoms of male hypogonadism, there might be an association of DHT levels within the setting of the normal range of total T concentrations in serum with these specific complaints. This would be a clinical picture similar to the side effects of 5ARIs [24], but not owing to this medication but due to other conditions. To assess this, we retrospectively reviewed patient charts in the electronic database Androbase [31] of the Center of Reproductive Medicine and Andrology (CeRA) in Münster (Germany).

## Materials and methods

### Study design and study population

We reviewed electronic health records of all men attending our institute (CeRA) for andrological complaints, presenting with erectile dysfunction, loss of libido or other signs associated with male hypogonadism (such as fatigue, depressed mood, lacking concentration) between January 1st, 2015 and October 31st, 2018. A total of 16,383 entries belonging to 5610 unique male patients were retrieved. To provide a real-life assessment, only the first visit of each patient was considered as single time-point for analysis. All subjects provided written informed consent to the analysis of serum and other material as approved by the Ethics Committee of the University and the State Medical Board (codes 2009-164-S; 2013-255-f-S). Patient-Reported Outcomes (PROs) concerning symptoms of erectile dysfunction and male aging symptoms were collected with the two validated questionnaires International Index of Erectile Function (IIEF-EF) and Aging Males Symptoms scale (AMS). The IIEF-EF and AMS questionnaires were completed by 738 and 807 subjects, respectively: however, as to reduce confounding factors, we excluded 461 subjects undergoing androgen replacement therapy, taking phosphodiesterase type 5 inhibitors or with either current or previous use of an aromatase inhibitor or 5ARI. In conclusion, 635 and 574 subjects were included in multinomial logistic regression analysis for AMS and IIEF-EF scores and hormones levels, respectively (Table 1). Albeit populations overlap, the analyses were performed independent of each other.

**Table 1:** Study population

	IIEF-EF cohort	AMS cohort	*P*
*N*	574	635	
Age (years)	47.3 ± 13.9	47.4 ± 13.8	0.828
BMI (kg/m^2^)	28.18 ± 5.36	28.11 ± 5.12	0.814
Total testosterone (nmol/l)	16.40 ± 8.74	16.25 ± 9.51	0.789
Free testosterone (pmol/l)	325.75 ± 210.20	325.94 ± 255.56	0.989
Estradiol (pmol/l)	90.92 ± 35.78	91.58 ± 35.28	0.753
DHT (nmol/l)	0.70 ± 0.37	0.69 ± 0.37	0.570
FSH (mIU/mL)	7.59 ± 9.86	8.02 ± 10.86	0.465
LH (mIU/mL)	4.12 ± 3.69	4.32 ± 4.68	0.428
AMS score	41.80 ± 13.24	41.65 ± 13.35	0.855
IIEF-EF score	16.06 ± 7.87	16.53 ± 8.95	0.356

Data expressed as mean ± SD

*P* calculated via Welch’s two-sample *t* test

### Endocrine assessment

All venous blood samples were obtained between 0800 and 1200 h. Serum was separated at 800 g. Samples were analyzed after collection or snap frozen and stored at − 20 °C.

Serum T and estradiol levels were measured by a commercial ELISA kit (DRG Instruments GmbH, Marburg, Germany). This immunoassay for T is calibrated quarterly against standards using liquid chromatography–mass spectroscopy (LCMS-MS), the immunoassays regularly pass this quality check and reproduce the results of mass spectroscopy with an imprecision of < 10% in the range for serum T concentrations between 5 and 20 nmol/L for T and for 25–300 pmol/L for estradiol. Intra-assay CVs were below 2%, mean inter-assay CVs below 5%.

Serum concentrations of SHBG, LH and FSH were determined using highly specific time-resolved fluoro-immunoassays (Abbott, Chicago, IL, USA). Mean intra-assay coefficients of variation (CV) were below 2% and mean inter-assay CVs below 5%.

Proteo-hormone assays are under quarterly blinded external quality control, as well and pass regularly.

Levels of free T (FTc) were calculated from levels of SHBG and total serum T according to the previously published calculations [32, 33].

A radio-immuno assay (RIA) was used to determine serum concentrations of DHT (Beckmann-Coulter, Krefeld, Germany). This is assay has 0.02% cross-reactivity with T after extraction and is calibrated against a standard of LCMS–MS with an imprecision of < 15% within the range of 0.1–5 nmol/l. Intra-Assay CV is 3.5%, mean inter-assay CV is 7%.

### Measurements of symptoms of male hypogonadism

The IIEF is a 15-item questionnaire developed by Rosen et al. in 1997 [34] and has since been considered one of the fundamental diagnostic tools for the evaluation of erectile dysfunction. The IIEF collects information pertaining to all domains of male sexual function: more in detail, the erectile function (EF) subdomain is investigated by questions 1, 2, 3, 4, 5 and 15, each one on a scale from 1 to 5, and has good sensitivity (0.97) and specificity (0.88) making it the most important part of assessment. Lower scores are associated with worse outcomes. While commonly described as a numeric scale, the IIEF-EF is better interpreted as a categorical variable with five categories (Table 2) [35].

**Table 2:** Classification of erectile dysfunction based on the International Index of Erectile Function—Erectile Function subdomain (IIEF-EF) scores

Severity of erectile dysfunction	IIEF-EF score
Severe	6–10
Moderate	11–16
Mild-to-moderate	17–21
Mild	22–25
No erectile dysfunction	26–30

Adapted from [35]

The AMS score, developed by Heinemann in 1999 [36], is a rating scale aimed to assess the prevalence and severity of subjectively perceived symptoms traditionally associated with male aging. 17 items are part of the AMS scale, each one on a scale from 1 to 5; in contrast to the IIEF-EF, worse outcomes are associated with higher scores. The scale is better interpreted as a categorical variable with four categories (Table 3) [37].

**Table 3:** Classification of symptoms of male aging, based on the Aging Males’ Symptoms (AMS) scores

Severity of complaints	AMS score
No or little complaints	17–26
Mild complaints	27–36
Moderate complaints	37–49
Severe complaints	50–75

Adapted from [37]

### Questionnaire collection

Questionnaires have been collected electronically using a web-based electronic multilingual PRO (ePRO) system (MoPat) [38]. Patients were provided with individual mobile devices and were allowed to answer all questions in a quiet, separate room while waiting for the medical consultation. The questionnaires have, thus, been completed by patients without the need of discussing with the clinician, therefore circumventing the possible reporting bias resulting from the direct interaction with a healthcare professional. Results are directly and electronically transferred into the patient database, eliminating transcription errors. Although first validated in a dermatology setting, the system has since been used in other areas, including andrology and reproductive medicine [38].

### Statistical analysis

Statistical analysis was performed on the statistical software R, version 3.5.1 [39]. Data cleaning was performed with the *tidyverse* package [40]. Normality of data distribution was assessed via the Kolmogorov–Smirnov one-sample test for goodness-of-fit; log-transformation was used for right-skewed distributions. Linear regression (for raw scores) and multinomial logistic regression (for AMS and IIEF-EF categories) models were devised in a two-step approach by performing univariate analysis, later followed by multivariate analysis for all relevant statistically significant parameters, with the polr function of the *MASS* package [41]. Significance was set at *p* < 0.05.

## Results

### Aging male symptom scale

**Fig. 1:**
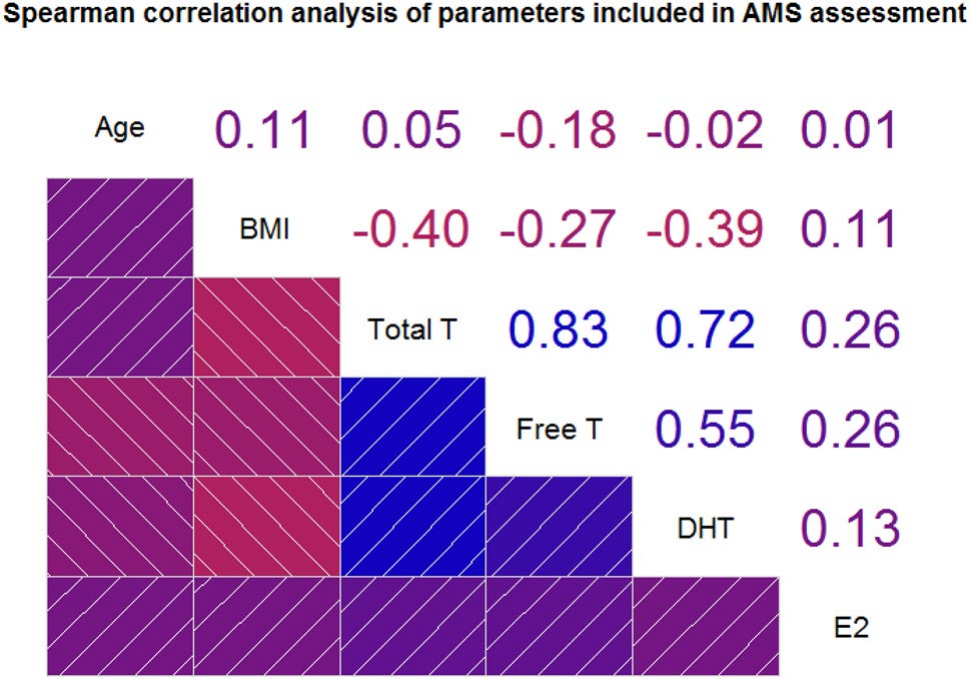
Spearman’s correlation matrix for parameters included in analysis of symptoms of male aging. Upper panel: rho coefficients for single bivariate correlation. Lower panel: negative correlation depicted in shades of red; positive correlation depicted in shades of blue

We performed statistical modeling using the AMS score as a dependent (outcome) categorical variable following the statistical approach described beforehand. To assess the risk of collinearity in the models, we performed correlation analysis using Spearman’s rho (Fig. 1). All androgens were positively correlated with each other with medium-large effect size (T vs FTc, rho = 0.83, *p* < 0.001; T vs DHT, rho = 0.72, *p* < 0.001; FTc vs DHT, rho = 0.52, *p* < 0.001). BMI had low-moderate negative correlation with all androgens (T, rho = − 0.40, *p* < 0.001; FTc, rho = − 0.27, *p* < 0.001; DHT, rho = − 0.39, *p* < 0.001); marginal effect size was observed for correlation between age and other variables included in analysis. Results of both univariate and multivariate analyses are reported in Table 4. In univariate analysis, DHT and T were the only analytes reaching statistical significance (respectively *p* = 0.0015 and *p* = 0.0414), whereas estradiol and FTc were removed from subsequent analysis. BMI and patients’ age did not reach statistical significance and were removed from subsequent analysis as well. A biochemical diagnosis of hypogonadism, defined as T < 12 nmol/l, was made in 219/645 (33.95%) subjects. The presence of hypogonadism was also included in the analysis and resulted statistically significant (*p* = 0.0051). Given the obvious bias resulting from introducing both T and hypogonadism in the same model, we performed two separate calculations, also described in Table 4. When assessing the interaction between DHT and hypogonadism, we observed a difference between hypogonadal and eugonadal subjects: no significant interaction for DHT was present in subjects with low T (OR 0.40, 95% CI 0.11–1.41, *p* = 0.1527); whereas in eugonadal subjects, higher DHT levels were significantly associated with less symptomatic AMS categories (OR 0.53, 95% CI 0.33–0.86, *p* = 0.0108). In regards to multivariate analysis for DHT and T, we found no significant effects for T (OR 1.00, 95% CI 0.98–1.02, *p* = 0.9283), but confirmed the role of higher DHT levels in favoring lower AMS scores in biochemically eugonadal men (OR 0.52, 95% CI 0.31–0.88, *p* = 0.0151). Mean T and DHT levels, stratified according to AMS categories, are reported in Supplementary Table 1. Additionally, we also used raw AMS scores to measure the effects of DHT and T levels in the two subgroups: a significant, negative effect was found for DHT levels in the eugonadal population (*β* = − 4.29, *p* = 0.0404), whereas no significant effects were found in the hypogonadal population, or for T in both subgroups (Supplementary Table 2).

**Table 4:** Parameters included in univariate (*panel A*) and multivariate (*panel B*) multinomial logistic regression analysis for symptoms of male aging, according to AMS scores. Hypogonadism: Total testosterone < 12 nmol/L

	Univariate analysis	
Parameters	OR [95% CI]	*p*-value
Panel A		
DHT (nmol/l)	0.53 [0.36–0.79]	**0.0015**
Presence of hypogonadism (yes)	0.66 [0.49–0.88]	**0.0051**
Total testosterone (nmol/l)	0.98 [0.97–1.00]	**0.0414**
Age (years)	1.00 [0.99–1.01]	0.6818
BMI (kg/m^2^)	1.05 [0.97–1.09]	0.5404
Estradiol (pmol/l)	1.00 [0.99–1.01]	0.4902
Free testosterone (pmol/l)	1.00 [0.99–1.01]	0.8057

Parameters	Multivariate analysis OR [95% CI]	*p*-value
Panel B		
Adjusting for interaction between DHT and hypogonadism		
DHT (nmol/l): Hypogonadism (yes)	0.40 [0.11–1.41]	0.1527
DHT (nmol/l): Hypogonadism (no)	0.53 [0.33–0.86]	**0.0108**
Adjusting for DHT and total testosterone		
DHT (nmol/l)	0.52 [0.31–0.88]	**0.0151**
Total testosterone (nmol/l)	1.00 [0.98–1.02]	0.9283

Significant results are highlighted in bold

### IIEF-EF scale

**Fig. 2:**
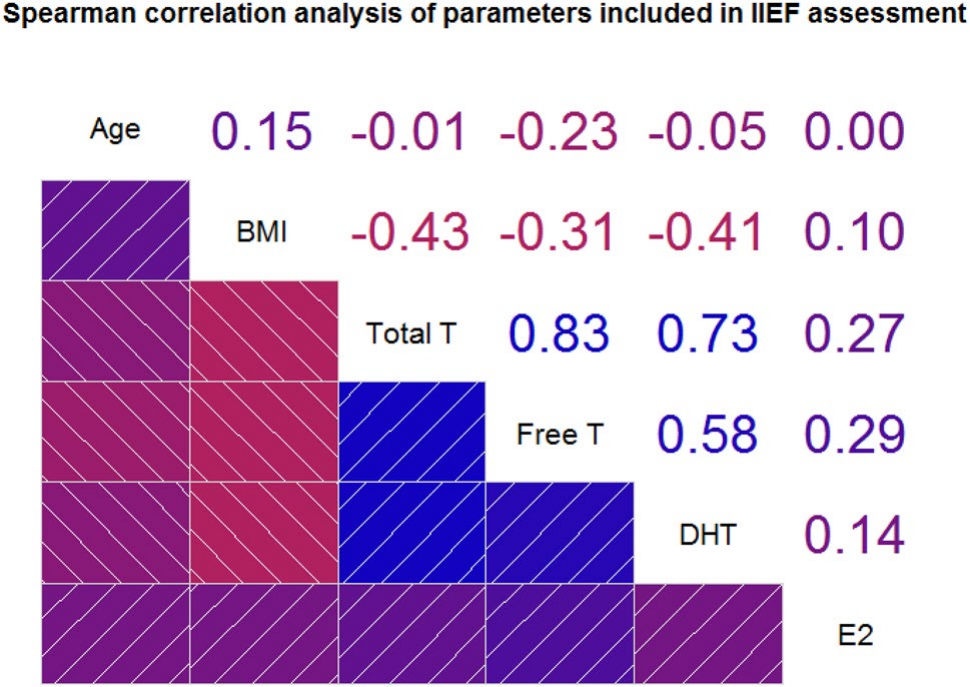
Spearman’s correlation matrix for parameters included in analysis of erectile function. Upper panel: rho coefficients for single bivariate correlation. Lower panel: negative correlation depicted in shades of red; positive correlation depicted in shades of blue

An approach similar to the one described above has been used to assess the effects of different parameters on the categorical classification of the IIEF-EF subdomain. Correlation analysis was performed to assess the relationship between variables to eventually prevent bias resulting from collinearity (Fig. 2); we reported, as expected, a high degree of correlation between T and FTc (rho = 0.83, *p* < 0.001) and between DHT and both T (rho = 0.73, *p* < 0.001) and FTc (rho = 0.58, *p* < 0.001). BMI was also inversely associated with all androgens, although with smaller effect size (T, rho = − 0.40, *p* < 0.001; FTc, rho = − 0.27, *p* < 0.001; DHT, rho = − 0.39, *p* < 0.001); whereas, patients’ age had marginal effects on all parameters included in analysis. Results of both univariate and multivariate analyses are reported in Table 5. Similar to the AMS analysis, DHT and T were the only two analytes reaching statistical significance (respectively, *p* = 0.0375 and *p* = 0.0193), whereas estradiol and FTc were removed from subsequent analysis having failed to reach statistical significance. The clinical status of hypogonadism was once again entered as a binomial variable and was kept for further analysis based on its significance (*p* = 0.0042); 188/574 (32,75%) subjects were considered hypogonadal based on their serum T levels. BMI and patients’ age were excluded as they failed to reach statistical significance. Following the same approach used in AMS analysis, we performed two separate calculations to separately describe the two models considering either serum T levels or the biochemical definition of hypogonadism (Table 5). However, different from what we describe regarding the AMS scores, no significant interactions were observed in regards to DHT levels in both eugonadal (OR 1.40, 95% CI 0.92–2.13, *p* = 0.1164) or hypogonadal (OR 0.85, 95% CI 0.35–2.04, *p* = 0.7188) subjects, as well in regards to DHT (OR 1.20, 95% CI 0.70–2.06, *p* = 0.4965) and T (OR 1.02, 95% CI 0.99–1.04, *p* = 0.1902) in the whole study population. Albeit, as the univariate analysis demonstrated inverse associations of both total T and DHT with IIEF-EF scores, it is likely that these hormones have an interdependent role in erectile function which is linear, unlike the non-linear association with AMS scores (see above). Mean T and DHT levels, stratified according to IIEF-EF categories, are reported in Supplementary Table 3**.** Linear regression analysis of raw scores failed to identify any statistically significant effect of either DHT or T on IIEF-EF scores (Supplementary Table 4).

**Table 5:** Parameters included in univariate (*panel A*) and multivariate (*panel B*) multinomial logistic regression analysis for erectile dysfunction, according to the EF domain of the IIEF. Hypogonadism: Total testosterone < 12 nmol/L

	Univariate analysis	
Parameters	OR [95% CI]	*p* value
Panel A		
DHT (nmol/l)	1.53 [1.02–2.29]	**0.0375**
Presence of hypogonadism (yes)	1.59 [1.16–2.18]	**0.0042**
Total testosterone (nmol/l)	1.02 [1.00–1.04]	**0.0193**
Age (years)	1.01 [0.97–1.04]	0.1081
BMI (kg/m^2^)	1.02 [0.99–1.06]	0.1096
Estradiol (pmol/l)	1.01 [0.99–1.01]	0.1117
Free testosterone (pmol/l)	1.01 [0.99–1.01]	0.1188

Parameters	Multivariate analysis OR [95% CI]	*p*-value
Panel B		
Adjusting for interaction between DHT and hypogonadism		
DHT (nmol/l): Hypogonadism (yes)	0.85 [0.35–2.04]	0.7188
DHT (nmol/l): Hypogonadism (no)	1.40 [0.92–2.13]	0.1164
Adjusting for DHT and total testosterone		
DHT (nmol/l)	1.20 [0.70–2.06]	0.4965
Total testosterone (nmol/l)	1.02 [0.99–1.04]	0.1902

Significant results are highlighted in bold

Hypogonadism: Total testosterone < 12 nmol/L)

## Discussion

Our findings support the view that symptoms of male aging might partly be explained by lower DHT concentrations, especially in subjects with serum T concentrations which are considered eugonadal. Our examinations elucidate that an actual increase of 0.1 nmol/l in serum DHT results in a 4.67% decrease in the odds of having more severe symptoms based on the AMS score in biochemically eugonadal men. This is quite in agreement with descriptions of the clinically relevant sexual side effects of DHT-lowering drugs (5-alpha-reductase-inhibitors) that keep T levels within the normal range [24].

Symptoms of male aging are often associated with hypogonadism [42–44]. These symptoms often require months, or even years of continuous androgen replacement therapy before significant improvements might be perceived [10], but especially sexual functions can be improved faster [45, 46]. These symptoms can also be associated with other endocrine parameters: erectile disorders might actually be the clinical manifestations of several conditions affecting other glands or be the result of cardiovascular impairment or damage of nerves as seen in, e.g., type 2 diabetes mellitus [47]. Also muscle weakness has been associated with an impairment in another byproduct of Leydig cells, INSL3 [48, 49], as well as with thyroid [50] and adrenal [51] disorders. Several endocrine pathways have been explored in these regards in the EMAS Study, suggesting a complex interplay between the psychological aspects of male sexual behavior, mostly regulated by E2, and the sexual functioning under more direct control from T [52]. Sexual desire is undoubtedly one of the symptoms most commonly associated with both aging and male hypogonadism [53]; however, some patients undergoing treatment with 5α aromatase inhibitors, such as finasteride, have experienced a dramatic loss of sexual drive, possibly affecting erectile and reproductive function as well [21, 54] without being hypogonadal in the classical sense of low T concentrations, which might partially be explained by the reduced serum DHT levels.

Sexual behavior—or rather, mating behavior—has been often studied in animal models, and stimulating effects were found in rats being administered DHT and estradiol into either the medial amygdala or the lateral septal regions. DHT has also been proven to promote the release of both pro- and anti-inflammatory cytokines [55] in adipocytes, suggesting another possible mechanism for some symptoms. Lower DHT levels might therefore provide an explanation for increased severity of symptoms in otherwise “healthy” subjects. Further support in these regards comes from clinical reports from subjects undergoing treatment with 5-alpha-reductase-inhibitors [56–58], who develop depression, fatigue and sexual symptoms within the classical eugonadal range of T concentrations.

Also, significant results were observed regarding erectile function: both T and DHT are associated with it. However, the results are not confirmed in multivariate approaches where these hormones were corrected for each other. While sexual symptoms assessed by the AMS score seem to be independently influenced by T and DHT and namely in a non-linear fashion (see above), this is not the case for erectile function. Both hormones seem to have a cooperative and linear effect on erection.

In summary, the symptoms measured by the AMS score seem to be influenced by T concentrations within the hypogonadal range and by DHT levels within the eugonadal range. Both hormones seem to act independently. The symptoms measured by the IIEF-EF score, however, exhibit a linear association with T and DHT concentrations and these hormones do not act independently in this case.

These findings suggest that androgens actually play a role in the multifactorial pathogenesis of erectile dysfunction, supporting earlier approaches demonstrating that the effect of erection-enhancing drugs such as PDE5 inhibitors require normal T levels [59] and that endothelial function is also dependent on adequate T levels [60].

More strongly, T and independently, DHT, play a pivotal role in controlling sexual desire [61]. The diagnostic work-up of patients’ symptoms of hypogonadism in general and especially with erectile dysfunction must include adequate medical, sexual and psychosocial history taking [62], as also suggested by recent guidelines [3, 63, 64]. Additionally, sexual desire should be considered in the wider context of the couple: several factors could be involved in a decline in sexual desire possibly independently of DHT levels, as commonly occurring in the “Couplepause” [65] or in the presence of other sexual dysfunctions, such as premature ejaculation [66, 67]. The presence of additional comorbidities such as diabetes [68, 69], or health-risk behaviors such as smoking, drinking alcohol or using drugs [70], should also be considered to adequately provide a tailored treatment, if needed, which extends beyond the mere use of medications. Whether these findings could be applied to specific forms of hypogonadism, such as Klinefelter Syndrome [71], is a question largely left unanswered and further studies are therefore warranted.

Compared to the EMAS population, which included non-institutionalized men aged 40–79 years, all subjects included in the present study had andrological complaints, such as erectile dysfunction, loss of libido and other symptoms of male hypogonadism. In this regard our study reflects a “real-life” setting, but with all subjects included in the analysis being independent of other comorbidities and free of co-medications.

## Limitations

This is a real-life study of patients attending a highly specialized center for treatment of andrological conditions, but despite the great number of subjects included in analysis, several limitations should be considered. As a retrospective cross-sectional study, this research project does not allow to come to definite conclusions concerning the role of DHT in the pathogenesis of selected symptoms of male aging. Furthermore, this study does not measure longitudinal trends in symptoms or in endocrine status.

A clinical trial aiming to assess the efficacy of exogenous DHT administration was effective [30] but more studies are required to understand whether treatment with either DHT (that would lack estrogenic activity and, thus, promote loss of bone mass) or rather additional T that would be 5-alpha-reduced and aromatized and, thus, contribute to higher DHT levels as well as higher estradiol concentrations might actually improve symptoms of hypogonadism. It also remains unclear what causes the marked differences of serum DHT concentrations within the eugonadal range of normal T levels. It may be speculated that this is caused by genetically modified expression of the 5-alpha-reductase isoenzymes. It is also possible that nutritional factors such as polyunsaturated fatty acids, zinc, and green tea as well as riboflavin influence 5-alpha-reductase activity [72, 73].

## Conclusions

The presence of symptoms of hypogonadism with biochemically normal levels of T but low concentrations of DHT is, according to our results, a matter for discussion.

Once biochemical hypogonadism has been excluded, DHT can be a useful addition to the endocrine assessment of subjects complaining of symptoms traditionally associated with androgen deficiency.

Our results contribute to the observations by which 5-alpha-reductase inhibitors can affect male sexual function even within the eugonadal range of T.

## Supplementary Information

Below is the link to the electronic supplementary material.

Supplementary file1 (DOCX 15 kb)
