# Supplementary material for: Serum concentrations of dihydrotestosterone are associated with symptoms of hypogonadism in biochemically eugonadal men
Source: J Endocrinol Invest. 2021 Apr 3;44(11):2465–74. doi: 10.1007/s40618-021-01561-0 (PMC8502125; doi:10.1007/s40618-021-01561-0)
Supplement: Supplementary file 1 — Supplementary file1 (DOCX 15 kb) [file 40618_2021_1561_MOESM1_ESM.docx]

**Supplementary table 1.** Mean ± SD for DHT and testosterone, stratified by severity of AMS category.

| **Aging Males Symptoms scale** | **No or little complaints (a)** | **Mild complaints** | **Moderate complaints** | **Severe complaints (b)** | p-value (ANOVA) | p-value (a vs b) |
| --- | --- | --- | --- | --- | --- | --- |
| **Hypogonadal men (T < 12)** |  |  |  |  |  |  |
| N | 20 | 49 | 79 | 71 |  |  |
| DHT (nmol/l) | 0.48 ± 0.29 | 0.45 ± 0.17 | 0.45 ± 0.23 | 0.40 ± 0.16 | 0.296 | 0.267 |
| Testosterone (nmol/l) | 7.48 ± 2.90 | 8.91 ± 2.47 | 8.60 ± 2.56 | 8.19 ± 2.76 | 0.165 | 0.335 |
| **Eugonadal men (T > 12)** |  |  |  |  |  |  |
| N | 57 | 126 | 124 | 109 |  |  |
| DHT (nmol/l) | 0.88 ± 0.325 | 0.84 ± 0.371 | 0.82 ± 0.37 | 0.78 ± 0.41 | 0.433 | 0.09 |
| Testosterone (nmol/l) | 20.6 ± 5.91 | 20.3 ± 6.81 | 20.9 ± 10.5 | 19.7 ± 11.3 | 0.799 | 0.53 |

**Supplementary table 2**. Linear regression analysis for symptoms of male aging, according to raw AMS scores. Hypogonadism: Total testosterone < 12 nmol/L).

| **Parameters** | β (Std. Error) | **p-value** |
| --- | --- | --- |
| DHT (nmol/l): Hypogonadism (yes) | -6.312 (5.093) | 0.2157 |
| DHT (nmol/l): Hypogonadism (no) | -4.289 (2.088) | **0.0404** |
| Hypogonadism (yes) (nmol/l):NormalTLow | 0.348 (0.310) | 0.2619 |
| Testosterone (nmol/l): Hypogonadism (no) | 0.080 (0.084) | 0.3422 |

**Supplementary table 3.** Mean ± SD for DHT and testosterone, stratified by severity of IIEF category.

| **IIEF-15 (EF domain)** | **No dysfunction (c)** | **Mild dysfunction** | **Mild-moderate dysfunction** | **Moderate dysfunction** | **Severe dysfunction (d)** | p-value (ANOVA) | p-value (c vs d) |
| --- | --- | --- | --- | --- | --- | --- | --- |
| **Hypogonadal men (T < 12)** |  |  |  |  |  |  |  |
| N | 47 | 26 | 24 | 35 | 56 |  |  |
| DHT (nmol/l) | 0.41 ± 0.19 | 0.45 ± 0.16 | 0.43 ± 0.15 | 0.46 ± 0.21 | 0.45 ± 0.25 | 0.825 | 0.382 |
| Testosterone (nmol/l) | 7.91 ± 2.74 | 8.56 ± 2.58 | 8.48 ± 2.3 | 8.73 ± 2.72 | 8.7 ± 2.68 | 0.575 | 0.144 |
| **Eugonadal men (T > 12)** |  |  |  |  |  |  |  |
| N | 51 | 39 | 66 | 101 | 129 |  |  |
| DHT (nmol/l) | 0.81 ± 0.38 | 0.84 ± 0.32 | 0.79 ± 0.290 | 0.86 ± 0.40 | 0.83 ± 0.37 | 0.846 | 0.740 |
| Testosterone (nmol/l) | 18.5 ± 5.35 | 20.8 ± 7.27 | 20.8 ± 10.9 | 20.9 ± 6.55 | 20.0 ± 8.49 | 0.47 | 0.147 |

**Supplementary table 4**. Linear regression analysis for erectile dysfunction, according to the raw score of the EF domain of the IIEF. Hypogonadism: Total testosterone < 12 nmol/L).

| **Parameters** | β (Std. error) | **p-value** |
| --- | --- | --- |
| DHT (nmol/l): Hypogonadism (yes) | 0.210 (3.259) | 0.949 |
| DHT (nmol/l): Hypogonadism (no) | -1.225 (1.276) | 0.337 |
| Hypogonadism (yes) (nmol/l):NormalTLow | -0.010 (0.203) | 0.962 |
| Testosterone (nmol/l): Hypogonadism (no) | -0.050 (0.057) | 0.375 |
